# Therapeutic Applications of Nanozymes in Chronic Inflammatory Diseases

**DOI:** 10.1155/2021/9980127

**Published:** 2021-08-11

**Authors:** Haojue Wang, Zichen Cui, Xuan Wang, Shui Sun, Dongsheng Zhang, Chuanyun Fu

**Affiliations:** ^1^School of Queen Mary, Nanchang University, Xuefu Avenue, Nanchang, Jiangxi 330031, China; ^2^Department of Joint Surgery, Shandong Provincial Hospital Affiliated to Shandong First Medical University, Jinan, Shandong 250021, China; ^3^Orthopaedic Research Laboratory, Medical Science and Technology Innovation Center, Shandong First Medical University & Shandong Academy of Medical Sciences, Jinan, Shandong 250117, China; ^4^Department of Stomatology, Shandong Provincial Hospital, Cheeloo College of Medicine, Shandong University, Jinan, Shandong 250021, China; ^5^Department of Stomatology, Shandong Provincial Hospital Affiliated to Shandong First Medical University, Jinan, Shandong 250021, China; ^6^School of Stomatology, Shandong First Medical University, Jinan, Shandong 250021, China

## Abstract

Since the discovery of horseradish peroxidase-like activity of magnetite nanoparticles in 2007, many researchers have investigated different types of nanoparticles that show enzyme-like activities, namely, nanozymes. Nanozymes possess high efficiency, stability, and low production costs compared to natural enzymes. Thus, nanozymes have already been widely studied in various domains including medical science, food industry, chemical engineering, and agriculture. This review presents the utilization of nanozymes in medicine and focuses particularly on their therapeutic applications in chronic inflammatory diseases because of their antioxidant-like activity. Furthermore, the treatment of chronic inflammatory diseases with nanozymes of different materials was introduced emphatically.

## 1. Introduction

Enzymes are biocatalysts with high efficiency that are highly selective to their specific substrates, but they also have some inherent defects such as low stability and high production cost, which limit their application [1]. Therefore, identifying enzyme mimics is an important study.

In 2007, it was discovered that magnetite nanoparticles possessed an activity similar to the enzyme-like property of horseradish peroxidases [2]. Further studies showed that these metallic nanoparticles exhibited an enzyme-like activity and were defined as nanozymes. These include nanoparticles that exhibit enzyme-like activities of peroxidase [3], catalase (CAT) [4], superoxide dismutase (SOD) [5], and hydrolase [6]. The most prominent characteristic of nanozymes is that the size of the nanoparticles is less than 100 nm in at least one dimension, which means they can be regarded as two-dimensional sheets with a high specific surface area and thus have excellent catalytic activities [7]. Moreover, the metallic nature of the nanozymes helps them in sustaining in hostile environments, such as high temperature, acidic environment, or alkaline environment; thus, nanozymes are more stable than natural enzymes [8]. Moreover, some nanozymes show unique characteristics and have low production costs compared to natural enzymes [9].

## 2. Application of Nanozyme in Medicine

### 2.1. Nanozyme-Based Sensors

#### 2.1.1. H_2_O_2_ Sensors

**Figure 1:**
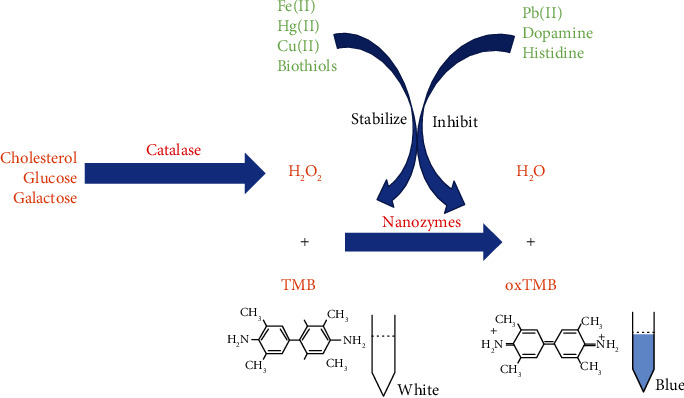
Brief diagram of the nanozyme-based H_2_O_2_ sensor and its application in the detection of cholesterol, glucose, and galactose.

Hydrogen peroxide is the most popular and detectable reactive oxygen species (ROS), which is formed by the reduction of oxygen. When the formation of ROS abnormally increases, H_2_O_2_ causes oxidative damage *in vivo* by targeting the DNA, proteins, and biofilms [10]. The sensing of H_2_O_2_ utilizes the nanozyme with peroxidase-like activity, such as iron-based nanoparticles (NPs) (with the detection limit of 50 *μ*M) [11], vanadium-based NPs (with the detection limit of 0.5 *μ*M) [12], and metal-organic framework-based nanocomposites (NCs) (with the detection limit of 0.24 *μ*M) [13] to catalyze the oxidation of 3,3′,5,5′-tetramethylbenzidine (TMB) by H_2_O_2_ to generate a blue-colored product, 3,3′,5,5′-tetramethylbenzidine (oxTMB) (Figure 1). Apart from this colorimetric reaction, scientists have also developed other methods for sensing H_2_O_2_ using an electrochemical process [14], fluorometric sensor [15], and Raman scattering sensor [16].

The sensing of H_2_O_2_ is quite important in the research of nanozyme, not only for the physiological effects of H_2_O_2_ but also due to the fact that detection of H_2_O_2_ is usually coupled with the sensing of other biomolecules or chemical particles such as biothiols [17], dopamine [18], metal ions [19–21], histidine, and Cu^2+^ [22], which can inhibit or stabilize the peroxidase-like activity of nanozymes and indirectly mediate the oxidative generation of TMB (Figure 1). Furthermore, some biomolecules like cholesterol [23], glucose [24], and galactose [25] can be oxidized by their oxidases to generate H_2_O_2_; so, combining these two reactions can help quantify the molecules easily (Figure 1). Due to the sensitivity of nanozymes to detect H_2_O_2_, it can also sense these molecules.

#### 2.1.2. Nanozyme-Based Enzyme-Linked Immunosorbent Assay (ELISA)

ELISA is a labelled immunoassay which is widely used in clinical laboratory to quantificationally detect antigens like cancer markers, proteins, viruses, and hormones. Traditional ELISA is based on the activity of natural enzymes and the unique interaction between the antigen and the antibody [26], but preparation and purification, as well as preservation of these enzymes, are expensive and laborious [27]. The development of nanozyme offers an alternative solution.

**Figure 2:**
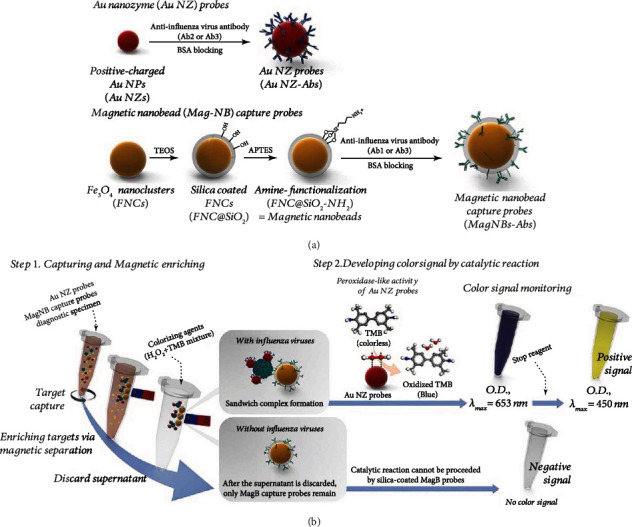
The preparation of the Au nanozyme probes and the magnetic capture nanobead probes and the procedure of this ELISA (cited from Scheme 1 of Oh et al. [28]).

Oh et al. developed an ultrasensitive ELISA to detect the influenza A virus [28]. This method utilizes magnetic nanobead capture probes and Au nanozyme probes to detect the influenza A virus. The Au nanozyme probes were made of Au NPs, which have higher stability and activity than traditional horseradish peroxidase, and anti-influenza virus antibodies (Ab2 or Ab3). The magnetic nanobead capture probes were made of magnetic nanobeads and anti-influenza virus antibodies (Ab1 or Ab3) (Figure 2(a)). Then, both of these were mixed, and they formed a sandwich structure with the influenza A virus; this structure was selected by a magnet. Then, the Au nanozyme probe with peroxidase-like activity catalyzed H_2_O_2_ to oxidize TMB into oxTMB (Figure 2(b)) [28].

Similar methods that use nanozymes with peroxidase-like activity (usually Au or Au alloy NPs) as the enzyme complex and TMB as the chromogenic substance are very useful for detecting different antigens. It has been employed for the detection of *Escherichia coli* [29], coronavirus [30], norovirus [31], and measles virus [32].

### 2.2. Nanozyme-Based Therapy

#### 2.2.1. Cancer Therapy

The application of nanozyme in cancer therapy can be classified into two categories. The first is a direct method, the basic principle of which is to generate ROS in tumor tissues utilizing the peroxidase-like activity and oxidase-like activity of nanozymes. Gao et al. designed a nanoplatform by integrating Au NPs and iron oxide NPs into dendritic mesoporous silica nanoparticles which served as the glucose oxidase to catalyze the oxidation of glucose to generate H_2_O_2_. Then, iron oxide converts H_2_O_2_ into high cytotoxic hydroxyl radicals by means of a Fenton-like reaction, which finally leads to the apoptosis of the cancer cells [33].

Radiotherapy and photodynamic therapy (PDT) are common therapeutics for cancer which are facilitated by the production of ROS [34]. But the generation of ROS needs oxygen, which is always deficient in cancer tissues [34]. So, the other method is to use catalase-mimic nanozymes to increase local oxygen concentration and indirectly improve the efficiency of radiotherapy and PDT to cancer tissues. Li et al. successfully synthesized porous platinum NPs which are catalase-mimic nanozymes that react with H_2_O_2_ and produce oxygen, which then effectively enhance the radiation dose in the targeted tumor tissues [35]. Similar effects were also reported by Hao et al. in PDT [36].

#### 2.2.2. Neuroprotection

Generation of ROS can be used in cancer therapy, but the overproduction of ROS is the pathological marker of many diseases, especially those that affect the neuro system. Prussian blue (PB) can effectively remove ROS due to its catalase-, superoxide dismutase-, and peroxidase-mimic activities. Based on these characteristics, Zhang et al. developed a hollow PB nanozyme which protects the neuro system against ischemic shock by scavenging ROS, relieving inflammation, and controlling cellular apoptosis [37].

#### 2.2.3. Anti-Inflammation

Because of the multienzyme-mimic activities, nanozymes display excellent ability in regulating oxidation-regulation reaction [37]. Inflammation is a pathological response that is intended to eliminate inflammatory stimuli and initiate tissue repair. Overproduction of ROS is an important characteristic of chronic inflammation, which leads to redox imbalance. Thus, restoring redox homeostasis is an important method to relieve inflammation reaction. Besides Alzheimer's disease and ischemic shock, nanozymes can also reduce the ROS level and resist inflammation in chronic inflammatory diseases.

Thus, we can summarize that nanozymes have been extensively studied in biomedical domains because of their unique characteristics. Herein, we will categorize nanozymes systematically and introduce their therapeutic applications in chronic inflammatory diseases.

## 3. Chronic Inflammatory Diseases

Chronic inflammation is a response to long-term inflammation, tissue injury, and attempted repair, which are usually caused by persisting infections, hypersensitivity diseases, and prolonged exposure to potentially toxic agents [38]. Typical chronic inflammatory diseases include Alzheimer's disease, Crohn's diseases, ulcerative colitis, rheumatoid arthritis, and periodontitis [39]. Inflammatory reaction is the collective characteristic of these diseases which includes vasodilation, increased vascular permeability, and leukocyte infiltration and activation [38]. The main method by which activated leukocytes recognize and eliminate the microbes or necrotic tissues is through phagocytosis, which mainly relies on ROS. H_2_O_2_, an ROS, then forms the H_2_O_2_-MPO-halide system, which is the most efficient sterilization system [38].

Although ROS as a physiological substance helps maintain hemostasis in normal condition, but the overproduction of ROS in chronic inflammatory reaction is highly toxic to normal tissue, its activity must be controlled *in vivo* by antioxidant mechanisms which involves enzymes like catalase, superoxide dismutase, and glutathione peroxidase [38]. The cause of most of the chronic inflammatory diseases is unclear, and so, the therapy for these diseases usually targets its inflammatory reaction [40]. Thus, the mimics of these enzymes may be potential drugs to relieve the inflammatory reaction and treat chronic inflammatory diseases.

An increasing number of researches have been performed toward the treatment of chronic inflammatory diseases. Using glucocorticoids to inhibit immune reaction and using nonsteroidal anti-inflammatory drugs (NSAID) to inhibit the biosynthesis of cyclooxygenase are the two major means used in clinical application to attenuate inflammation, which have extensive side effects of gastrointestinal complications, cardiovascular complications, and inducing or aggravating infection, among others [41–45]. Hence, identifying other anti-inflammatory agents is of utmost importance. Among these, antioxidants are widely sought after, and quite a few of antioxidants such as curcumin [46], polyphenol [47], chlorogenic acid [48], and ascorbic acid [49] have been reported for their anti-inflammatory potential. Some of these have already been used in clinical therapy. Thus, nanozyme, as the high-efficiency mimic of antioxidant enzyme, is no doubt a considerable option for chronic inflammatory disease therapy.

## 4. Therapeutic Applications of Nanozymes in Chronic Inflammatory Diseases

### 4.1. Cerium Oxide-Based Nanomaterial

Cerium oxide-based nanozymes are widely used in many fields, such as medicine, because of their unique characteristics. Cerium oxide NPs have superoxide dismutase-, catalase-, and oxidase-like activities [50, 51]. Furthermore, cerium oxide-based nanozymes are stable in both acidic and basic conditions. At physiological pH values (pH 7.4), cerium oxide-based nanozymes perform great SOD- and CAT-like activities; thus, the nanozymes can protect cells from oxidants, but under acidic conditions (pH 4.5), the oxidase-like activity of cerium oxide-based nanozymes can effectively kill cancer cells by producing ROS [52].

Some studies showed that the Ce^3+^/Ce^4+^ ratio depends on the CAT- and SOD-like activities, and cerium oxide nanoparticles can simulate the chemical reaction and antioxidant activity of CAT and SOD [53]; thus, it can protect cells by regulating the level of ROS in cells.

One of the important functions of cerium oxide nanoparticles is inhibiting inflammatory mediators and protecting the cell structure from inflammatory diseases. Inflammation *in vivo* and *in vitro* can be effectively treated by scavenging free radicals or ROS [54]. Arya et al. [55] synthesized spherical cerium oxide nanozymes as an anti-inflammatory drug to evaluate the protective effect of hypoxia on the lung. After repeated intraperitoneal injection, CeO_2_ was deposited in the lung, which reduced the oxidation of active oxygen and lipid, thus protecting the lung from oxidative stress and tissue damage caused by the endotoxin.

**Figure 3:**
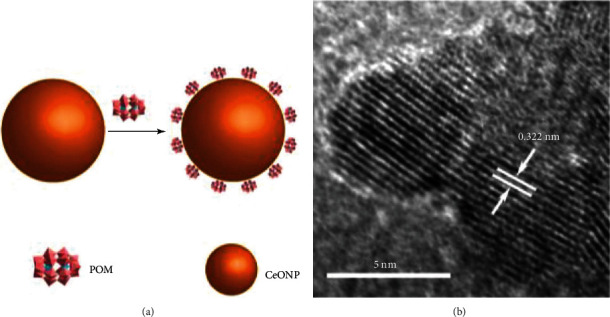
(a) Schematic structure of ceria/polyoxometalate hybrid molecule. (b) Electron microscope image of ceria/polyoxometalate hybrid molecule (cited from Figure 1 of Guan et al. [56]).

Studies have shown that the ceria nanozyme is very effective in neuroprotection. Alzheimer's disease is an insidious disease characterized by progressive neurodegenerative changes including amyloid-beta peptide aggregates, overproduction of ROS, and inflammatory reactions. Based on these pathological markers, Guan et al. [56] designed a ceria/polyoxometalate hybrid with SOD and proteolytic activities (Figure 3). This nanozyme could cross the blood-brain barrier, degrade amyloid-beta peptide aggregates, reduce the ROS levels, boost the proliferation of PC12 cells, and inhibit the activation of BV2 microglia cells induced by the amyloid-beta peptides. Thus, it provides an evidence for the therapeutic application of nanozymes in Alzheimer's disease.

Scientists have also developed an effective anti-ischemic treatment agent based on monodispersed ceria nanoparticles, which are loaded with edaravone and modified with angiopep-2 and poly (ethylene glycol) on their exterior [57]. This material can effectively treat stroke by eliminating ROS, as it can greatly enhance the brain uptake; simultaneously, it can effectively protect the blood-brain barrier and thus has great potential in stroke treatment.

Parkinson's disease is a hypokinetic disorder disease, which is characterized by the failure of the disinhibition that is normally mediated by the basal ganglia. The direct cause of Parkinson's disease is the loss of the substantia nigra pars compacta due to cell death. Kwon et al. [58] used CeO_2_ to enhance the activities of SOD and CAT enzymes and remove ROS inside and outside the cells to prevent the initiation of microglia and peroxidation of lipid and to protect tyrosine hydroxylase to treat Parkinson's disease.

As mentioned above, these experiments present the excellent ROS-reducing ability of ceria NPs inside and outside the cells, which greatly alleviates the oxidative stress in the brain and lung [55–58]. Curative effects of ceria NPs are shown in animal models with Parkinson's, stroke, and lung inflammation. Thus, the broad potentials of ceria NPs are shown in anti-inflammation therapy. But what needs consideration is how the ceria NPs are eliminated from the body, if the ceria NPs possessed biological toxicity, and also the stability and activity of this nanozyme. Hence, deeper research on ceria NPs is still needed.

### 4.2. Iron Oxide-Based Nanomaterial

Iron oxide-based nanozymes are great peroxidase mimics with excellent stability. Even when placed in high temperatures of 90°C and pH value from 1.5 to 12.0 for 2 hours, iron oxide-based nanozymes can still retain its catalytical activity, which is much better than the natural enzyme, which rapidly loses its activity at 40°C and pH value of 5.0 [59].

At present, iron oxide-based nanozymes have certain value in the treatment of chronic inflammatory diseases, but their usage as antioxidants has therapeutic effects in nervous system diseases [60]. *In vivo*, researchers demonstrated that iron oxide NPs protect cells from oxidative stress and apoptosis induced by H_2_O_2_. Also, iron oxide NPs can relieve intracellular oxidative stress, postpone animal aging, and prevent neurodegeneration because of their catalase-like activity. *In vitro*, the level of ROS of PC12 cells, which were exposed to MPP+, declined greatly due to the treatment of iron oxide NPs, and the death of the PC12 cells was alleviated. Therefore, the function of iron oxide NPs in the therapy of Parkinson's disease has been evaluated [60].

In addition, researchers used the Drosophila Alzheimer's disease model to examine the effects of iron oxide NPs on neuronal dysfunction. They found that dietary iron oxide NPs can improve neurodegeneration, due to the decomposition of ROS by iron oxide NPs. Another study developed a ferritin nanozyme (Fenozyme) formed by recombinant human ferritin (HFn) protein shells [61]. This nanozyme can not only specifically target blood-brain barrier endothelial cells (the effect of HFn) but also exhibits catalase-like activity to scavenge ROS. An *in vivo* experiment showed that Fenozyme significantly improved the destruction of the blood-brain barrier caused by parasites and significantly promoted the survival of infected mice.

In addition, it has been verified that iron oxide NPs have a protective effect on cardiomyocytes, because they have catalase- and peroxidase-like activities and are used in antioxidant therapy. Xiong et al. [62] reported that Fe_2_O_3_-DMSA NPs can preserve the heart from ischemic injury by inhibiting intracellular ROS in several ways and relieve the peroxidation injury of the membrane lipid.

The above experiments preliminarily demonstrate the peroxidase- and catalase-like activities of iron oxide NPs, which can effectively eliminate superfluous H_2_O_2_ generated under oxidative stress or produced through SOD and also successfully protect myocardial and neural cells [60–62]. An even better curative effect was observed in mice with myocardial ischemia and reperfusion, which were treated with iron oxide NPs compared to when the same model was treated with verapamil. No apparent cytotoxicity was observed in iron oxide NPs with the dosage between 0.01 and 0.5 mg ml^−1^.

### 4.3. Manganese-Based Nanomaterial

MnO_2_ NPs have been proven to have a variety of enzyme-like activities such as peroxidase-, oxidase-, catalase-, SOD-, and glutathione peroxidase-like activities, and they are more stable than natural enzymes. Therefore, MnO_2_ NPs have broad applications in different aspects, such as biotechnology, bioassays, and biomedicine [63]. Due to the abnormal increase in ROS level, the balance of redox *in vivo* will be destroyed, which will cause oxidative stress, and ultimately leads to the destruction of the structure and function of cellular macromolecules. The peroxidase- and SOD-like activities of nanozymes are generally utilized to control the level of ROS in cells, and this function plays an important role in protecting the cells.

**Figure 4:**
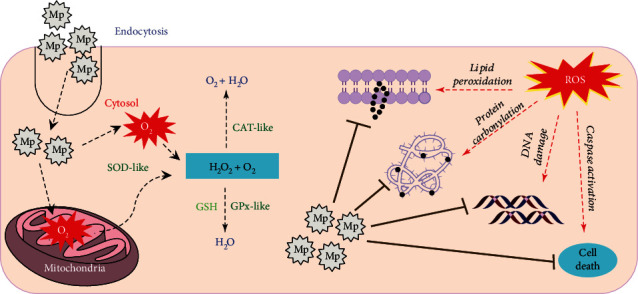
The basic mechanisms of the working of SOD-, CAT-, and GPx-mimic nanozymes in ROS-imbalanced cells (cited from the graphical abstract of Singh et al. [65]).

Singh et al. [64] found that Mn_3_O_4_ NPs with flower-like morphology performed the activities of several antioxidant enzymes such as SOD, CAT, and glutathione peroxidase. These activities are related to the mixed oxidation states of manganese, unusually large-sized pore, high stability against irreversible oxidation, and large surface area. They have proven that the multienzyme mimic, Mn_3_O_4_ nanoflowers, could modulate the redox status of the cells caused by oxidative stress by protecting biomolecules from protein oxidation, lipid peroxidation, and DNA damage mediated by ROS (Figure 4). Furthermore, these nanozymes, with the ability to mimic the cellular antioxidant enzymes to protect cell from oxidative damage, will not alter the response of Nrf2 protein under oxidative stress, so the nanozymes can regulate the redox homeostasis of cells without interfering with the antioxidant proteins/enzymes in the cells [65]. Also, the Mn_3_O_4_ nanoflowers showed great ability to save the cells from ROS-mediated inflammatory injury and prevent neurological diseases, such as Parkinson's, caused by the imbalance of ROS.

Besides the multienzyme-mimic activity, the high catalytic capacity of Mn_3_O_4_ NPs, compared to not only the natural enzyme but also other NPs, is an amazing finding. Mn_3_O_4_ NPs showed better ability in the scavenging of oxygen radical and hydroxyl radical when compared to Fe^2+^ and CeO_2_ NPs; thus, Mn_3_O_4_ NPs exhibited greater anti-inflammatory ability. Based on this, Yao et al. designed a strategy to cure ear inflammation in mice [66].

The multiple enzyme-mimic activities and the comparative high catalytic activities are the major characteristics of manganese-based nanomaterial, which are rarely found in other nanozymes [63]. This implies that manganese-based nanomaterials can protect cells from ROS-induced cell injury and inflammatory reaction without disturbing the inherent antioxidation system of the cells. Thus, manganese-based nanozymes can be a good choice for the treatment of chronic inflammation with broad prospect.

### 4.4. Gold- and Platinum-Based Nanomaterials

Gold nanomaterials have entered public sight as a nanozyme material because of the function of mimicking oxidase. In order to play a better role in simulating enzyme activity, some studies have used gold nanoparticles along with other materials to synthesize nanostructures, such as Au-Pt.

In the field of cell protection, gold nanozyme showed great antioxidant properties and highly catalytic activity. Keratinocytes are most affected by ultraviolet (UV) light irradiation, because these cells are located in the outermost layer of skin. UV-induced cell damage is caused by superfluous ROS. Xiong et al. [67] prepared enzyme-mimic Au-Pt NCs to catalyze the clearance of ROS. They have demonstrated that the active and biocompatible Au-Pt NCs can eliminate ROS induced by UV rays and prevent subsequent oxidative damage to cells *in vivo*.

### 4.5. Platinum-Based Nanomaterial

The major utilization of platinum in medicine is in anticancer therapy such as cisplatin, carboplatin, and oxaliplatin. With increased research in metal NPs, researchers have identified its excellent ability in anti-inflammation. Similar to other nanozymes, platinum NPs (PtNPs) performing as SOD and CAT mimics can eliminate ROS and reduce oxidative stress. Furthermore, PtNPs are different from other enzymes as they can directly inhibit the overproduction of nitric oxide, tumor necrosis factor-*α*, interleukin-6, and other inflammatory mediators; they can also inhibit the activity of macrophages through inhibition of the NF-*κ*B signaling pathway. Based on this comprehensive anti-inflammation ability, oral PtNPs produced a marked effect in mouse colitis which greatly alleviated local and systemic inflammatory reaction [68]. Similar effects were also observed in the prevention of pneumonia in mice exposed to smoke [69].

Although PtNPs are great antioxidants, they can be easily oxidized in air, but combining them with PdNPs can solve this problem and broaden the application of PtNPs. Shibuya et al. [70] used a mixture of PdNPs and PtNPs, namely, PAPLAL, to treat chronic diseases. Besides, due to the SOD- and catalase-like activities of PAPLAL, it can inhibit the intrinsic superoxide *in vivo* and treat aging-related skin diseases caused by oxidative stress.

### 4.6. Carbon-Based Nanomaterial

Carbon-based nanomaterials have been widely used in biomedicine. Compared to natural enzymes, carbon nanomaterials have higher stability and lower cost.

Fullerene is the first carbon-based nanozyme that has been found to possess SOD-like activity. C3 is a first-in-class functionalized water-soluble fullerene, and it has been confirmed that C3 can reduce ROS, which has been shown to be related to neurodegenerative diseases *in vitro*. Dugan et al. [71] found that after the treatment of the potent antioxidant C3, Parkinsonian motor ratings significantly improved in monkeys and the striatal dopamine levels increased. Even though the damage process had begun, C3 could reduce striatal injury. Furthermore, C3 has anti-inflammatory properties which can reduce MPTP- and 6-hydroxydopamine-induced neuronal cell death by reducing oxidative damage. These studies found little evidence of toxicity for C3.

### 4.7. Summary

Natural enzymes require strict physiological conditions for performing catalytic functions. Their limited stability in harsh environmental conditions and other significant drawbacks such as the high cost of synthesis, isolation, and purification greatly limit their practical applications [72]. Therefore, to address the limitations of natural enzymes, nanozymes have attracted considerable interest over the past decade owing to their obvious advantages. Nanozymes are nanomaterials with intrinsic enzyme-like characteristics which can offer unflinching biocatalytic activity even in the extreme conditions of pH, temperatures, and resistance to the digestion from proteases. Their advantages also include low cost, easy large-scale production, high stability, and smooth surface modification of nanomaterials [2, 72]. Moreover, the unique physicochemical properties of nanomaterials not only endow nanozymes with multiple functionalities but also provide more possibilities for rational design and future applications. As an alternative to natural enzymes, the antioxidant effects of nanozymes have been extensively explored for antiaging, anti-inflammatory, antioxidative, and neuroprotective functions and in the treatment of Alzheimer's and Parkinson's disease [73].

Although it is well established that nanozymes possess several distinct advantages over natural enzymes, they still face several limitations. Biological enzymes are highly selective to their targets; however, nanozymes show limited selectivity toward their substrates. So far, most studies focused on the activity regulation and only a few on their selectivity [74].
